# The Impact of Cardiac Rehabilitation on Psychosocial Factors, Functional Capacity, and Left Ventricular Function in Patients with Coronary Artery Disease: Systematic Review and Meta-Analysis

**DOI:** 10.12688/f1000research.151834.1

**Published:** 2024-06-05

**Authors:** Ali Suleiman Harbi, Dr Kim Lam Soh, Dr Putri Binti Yubbu, Kim Geok Soh

**Affiliations:** 1 Department of Nursing, Universiti Putra Malaysia, Serdang, Selangor, 43400, Malaysia; 2 Department of Pediatrics, Universiti Putra Malaysia, Serdang, Selangor, 43400, Malaysia; 3 Department of Sport Studies, Universiti Putra Malaysia, Serdang, Selangor, 43400, Malaysia

**Keywords:** Cardiac rehabilitation; Psychosocial factors; Functional capacity; Left ventricle

## Abstract

**Background:**

Cardiac rehabilitation is a multifaceted program implemented after adverse events. It aims at facilitating the return to normal life. This review aimed to examine the impact of cardiac rehabilitation on psychosocial risk factors, functional capacity, and left ventricular function.

**Methods:**

The following databases: CINAHL, Scopus, PubMed, and Cochrane Library have been searched to retrieve the randomized controlled trials that investigate the effectiveness of cardiac rehabilitation versus usual care on anxiety, depression, peak oxygen consumption, six-minute walk distance, left ventricular ejection fraction, and left ventricular end-systolic and diastolic dimensions. Filters were set to retrieve trials that were published in the English Language between 2000 and 2024. Risk of bias was assessed using the Cochrane risk-of-bias tool (Rob2). Data were analysed meta-analytically.

**Results:**

Twenty two (22) trials were included, randomized 2283 participants. A significant improvement favouring intervention groups was observed in anxiety SMD = -0.3890 (95% CI: -0.5640 to -0.2140; p˂0.001), depression SMD = -0.4032 (95% CI: -0.7114 to -0.0950; p= 0.002), peak oxygen consumption MD = 1.2471 (95% CI: 0.3963 to 2.0979; p = 0.004), six-minute walk distance MD = 36.0290 (95% CI: 7.7497 to 64.3082; p = 0.013), and left ventricular ejection fraction MD = 3.0650 (95% CI: 1.1279 to 5.0022; p = 0.001), Although cardiac rehabilitation had no significant effect in decreasing left ventricular end-diastolic dimension MD = -0.0480 (95% CI: -0.2609 to 0.1648; p = 0.658) and left ventricular end-systolic and MD = -0.0670 (95% CI: -0.2829 to 0.1489; p = 0.543) a favourable trend toward intervention group was seen. Risk of bias was high in 1 study and unclear in 7 studies.

**Conclusion:**

For patients with Coronary Artery Disease, cardiac rehabilitation demonstrated effectiveness in improving psychological symptoms such as anxiety and depression, functional capacity as measured by peak oxygen consumption and six-minute walk test, and left ventricular function.

## Introduction

Cardiovascular disease (CVD) remains the main cause of morbidity and mortality internationally, contributing to a significant health burden worldwide. Nearly 19 million deaths were associated with CVD in 2020, representing an 18.7% growth from 2010, and over 500 million individuals globally are affected by CVD. Coronary artery disease (CAD) is the most frequently encountered CVD and is associated with a decline in quality of life. ^1^^,^^2^ Despite technological progression in CAD treatment, patients still experience subsequent cardiovascular events, ^3^^,^^4^ resulting in frequent readmissions, and escalating the personal and social burden of CAD. ^5^ Hence, presence of ongoing management after being diagnosed with CAD is vital.

Cardiac rehabilitation (CR) is a multifaceted program implemented after an adverse event. It aims at facilitating the return to normal life and improving exercise capacity. ^6^ Cardiac rehabilitation includes health education, behavioural counselling, lifestyle management and exercise training. ^6^ It is recommended to start the program early in-hospital (phase I) and then continue with (phase II and III) either in-home or centre-based program. ^7^

The Presence of psychosocial risk factors such as anxiety and depression disturb the prognosis of CAD. ^8^^–^^11^ Studies showed that depression and anxiety increase the risk of frequent cardiac events and cardiac and all-cause mortality in patients with CAD. ^11^^–^^13^ Conversely, psychosocial well-being has been described as a protective factor. ^14^ Psychosocial risk factors adversely affect cardiac outcomes by encouraging an unhealthy lifestyle and by diminishing the likelihood of successful alteration of cardiac risk factors. ^15^ They are also associated with decreased adherence to medical treatment and rehabilitation. ^15^^–^^17^

As a principal clinical outcome of CR, Functional Capacity is a key factor in secondary prevention of CVD. ^18^ it refers to the capability of an individual to undertake aerobic work. ^19^ The collaborative performance of cardiovascular, pulmonary, and musculoskeletal systems determines an individual’s functional capacity. ^20^ Several studies have reported that the evaluation of functional capacity offers essential diagnostic and prognostic information about patients with CVD. ^21^ Therefore, it is imperative to assess the impact of CR on functional capacity. Functional capacity is frequently measured by exercise tests such as peak oxygen consumption (VO _2peak_) and 6-minute walk test (6-MWT). ^22^^,^^23^

The left ventricle is a core component of the cardiovascular system. The primary function of the left ventricle is to provide other organs with blood flow. Maintaining sufficient blood supply needs effective contractility of the left ventricle. Patients with CAD may experience cardiac remodelling; a process defined as a group of alterations within the heart that lead to changes in the size, shape and function of the heart. ^24^ The progression of cardiac remodelling may lead to severe left ventricle impairment and chronic heart failure. ^25^ Therefore, the evaluation of cardiac rehabilitation on left ventricular function is very important.

Cardiac rehabilitation research is a dynamic field, and the presence of new research comparing the impact of CR vs usual care necessitates incorporating recent findings, analysing the growing body of literature, and recognizing the potential source of heterogeneity. The aim of this systematic review and meta-analysis was to examine the impact of CR on specific psychosocial risk factors functional capacity, and left ventricular function (i.e. anxiety and depression, 6-MWT, VO _2peak_, left ventricular ejection fraction (LVEF), left ventricular end-diastolic dimension (LVEDD), and left ventricular end-systolic dimension (LVESD)).

## Methods

### Search strategy

The current systematic review and meta-analysis adhered to the Preferred Reporting Items for Systematic Reviews and Meta-Analysis (PRISMA) guidelines. ^26^ Two authors (ASH and PY) independently searched the following databases: PubMed, CINAHL, Scopus, Cochrane Library and EMBASE. Additionally, the references of included studies were manually searched to retrieve studies undetected in the primary search. Medical Subject Headings (MeSH) were used in the search process to retrieve the relevant studies. The filters were set to retrieve studies that were published in the English Language between 2000 and 2024. The retrieved studies were checked primarily by titles for potential inclusion in this review, subsequently, those potentially included papers were checked by abstract and then full text for eligibility. Any conflict was resolved by discussion until consensus was reached, and the final decision about the included and excluded paper was made by the senior author (KLS).

### Inclusion and exclusion criteria

This systematic review and meta-analysis included studies that met the following criteria: 1) randomized controlled trial; 2) examined the impact of CR on at least one outcome of interest; 3) involving patients with CAD aged ≥ 18 years; 4) the minimum follow-up duration of four weeks. studies that did not meet these criteria were excluded. In addition, studies with mixed populations (i.e., patients with CAD and patients with non-CAD) were excluded. The EndNote software (Endnote X9) was used to remove the duplicated articles (alternatively many reference managers can be accessed and used freely such as Mendeley Reference Manager) then a manual check was done.

### Data extraction and analysis

A pre-set Microsoft Excel spreadsheet was prepared to record the extracted data. Two authors (ASH and PY) independently extracted the following data: first author’s name; year of publication; the location where the study was conducted; sample size and the number of participants in each group; percentage of each gender; intervention’s main components; duration of follow-up; populations’ diagnosis; the p-value for each compression; and post-intervention means and standard deviations for each group.

The population, intervention, comparator and outcomes (PICO) framework was used to guide this review. The intended population encompassed patients with CAD, including those who were diagnosed with, myocardial infarction, and those who had undergone percutaneous intervention, or coronary artery bypass graft, The considered intervention was the home-based CR (HBCR) or centre-based CR (CBCR); the comparator was those patients who received the usual or no care, the outcomes of interest are: 1) psychosocial risk factors measured by anxiety and depression; 2) functional capacity measured by VO _2peak_ and 6-MWT; and 3) left ventricular function measured by left ventricular ejection fraction (LVEF), left ventricular end-diastolic dimension LVEDD, and left ventricular end-systolic dimension LVESD.

Data were analysed following the guidelines of the Cochrane Handbook for systematic reviews of interventions. ^27^ Using Jamovi software (Jamovi 24.11). A random effect statistical model was applied. To estimate the effect of CR versus usual care, post-intervention between groups mean difference (MD) and standard deviations were used. Alternatively, standardized mean difference (SMD) was used in case the outcome was measured by different scales among the studies. Heterogeneity was tested using I ^2^, whereby values of 25%, 50%, and 75% indicate low, moderate, and high heterogeneity, respectively. Hypothesis testing was performed at a two-tailed 0.05 level and a 95% CI. If I ^2^ values showed high heterogeneity (I ^2^ > 50%), sensitivity analysis was performed by using ‘leave-one-out approach, removing one study at a time and observing the impact on heterogeneity. Publication bias was assessed visually by a funnel plot and statistically by the Egger test. Subgroup analysis was performed based on the duration of follow-up (≥ 3 vs ˂ 3 months), and population average age (≥ 60 vs ˂ 60 years).

### Risk of bias

Two authors (ASH and PY) independently assessed the risk of bias in the included studies. Version 2 of the Cochrane risk-of-bias tool for randomized trials (RoB 2) was employed to assess the risk of bias in the included trials. ^28^ RoB 2 is structured into a set of domains of bias: (1 bias arising from the randomization process; (2 bias due to deviations from intended interventions; (3 bias due to missing outcome data; (4 bias in the measurement of the outcome; (5 bias in the selection of the reported result. For each domain, a set of questions (signalling questions) is designed to guide the user in extracting information related to the risk of bias. Judgment regarding the risk of bias arising from each domain is made by an algorithm, based on answers to the signalling questions. Judgment can be ‘Low’ or ‘High’ risk of bias, or ‘Some concerns’. the final decision about the risk of bias Judgment was made by the senior author (KLS).

### Certainty of evidence assessment

The quality of evidence across the outcomes was assessed using the Grading of Recommendation Assessment, Development and Evaluation (GRADE) approach. ^29^ GRADE has four levels of evidence: high, moderate, low, or very low. Based on GRADE guidelines RCTs are considered high-quality evidence, then they may be downgraded 1 or 2 levels based on the assessment of Risk of bias, inconsistency, indirectness, imprecision, and publication bias for each outcome.

## Results

### Search results

**Figure 1.**
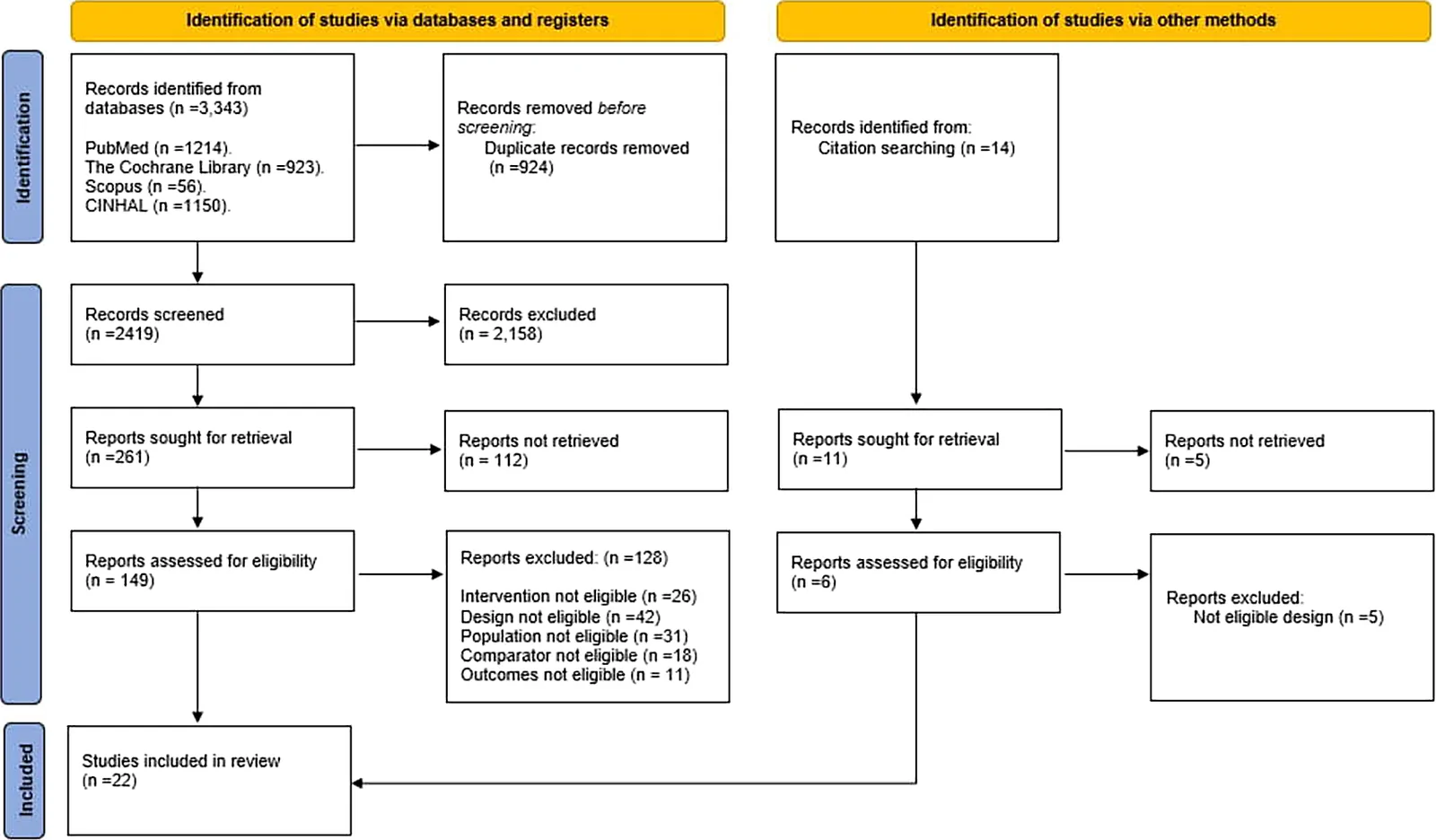
PRISMA flow chart.

The preliminary search in the databases identified 3,357 studies. Duplicated articles were removed using the EndNote software. The remaining articles were screened by titles for potential inclusion. The retrieved articles were checked by reading the abstracts. After excluding the articles that did not meet the inclusion criteria. 95 articles were eligible for full-text reading. The final number of trials included in this review was 22 studies. The details of the search process are illustrated in Figure 1.

### Characteristics of included studies

The present systematic review and meta-analysis includes 22 RCTs, ^22^^,^^30^^–^^50^ conducted in 11 countries, 5 studies each in Iran and China, 2 studies each in Portugal, Japan, and Denmark, and 1 study each in USA, Brazil, Hong Kong, Turkey, Finland, and Indonesia. The included trials were published between 2003 and 2023. randomized 2283 participants 1196 in intervention groups and 1087 in control group. Sample sizes ranged from 29 to 310, and the mean of participants’ ages ranged from 40 to 85 years. One study reported a high percentage (71.2%) of female participants, while the rest of the studies exhibited low female representation, and the durations of follow-up ranged from 4 weeks to 12 months. Table 1 illustrates the studies’ characteristics.

**Table 1.** Characteristics of included studies.

Authors Year country	Sample size		Age year	Follow-up duration	Population	Intervention main components	Outcomes	Between groups p value
	Exp	Cont						
	Female (%)							
(Sharif et al., 2012) Iran.	40	40	40-70	2- month	patients scheduled for CABG	Two sessions/week 2 hours each of education and training include **:** • Medication. • Diet. • Weight control. • Stress management. • Quitting smoking. • Light physical exercise • Relaxation.	Anxiety	0.079
	(70%)						Depression	0.014
(Snoek et al., 2021) Finland.	58	60	60-59	12- month	Patients with CAD	• Remote monitoring and coaching using a smartphone and Bluetooth-connected heart rate belt. • The device records training mode, training time and training intensity. • Participants were advised to exercise at least 30 min/day for 5 days/week. • participants had insight into their training history through an individual webpage. • A standard operating procedure, composed with a psychologist, was used for the individual coaching. • They were contacted weekly by telephone for supportive guidance.	Anxiety	0.11
	NP						Depression	0.24
							Vo _2peak_	0.74
(Xu et al., 2016) China	26	26	55.5-55.8	4-week	Patients with CAD	*1-Inpatient phase for 1 week,* • Casual limb movements while in bed and simple walk training. • Energy expenditure estimated at 2 to 4 (METs). • Intensity targeted 4 and 5 perceived exertion Borg (PEB) scale rating. • Reach 60% (HRmax). *2-Outpatient phase for 4 weeks* • A 5-minute warm-up (stretching), 20-minute aerobic exercise (walking or jogging, gymnastics), and a 5-minute cool-down (stretching). • Brochure that included information about cardiac disease, risk factor management, the importance of exercise, dietary advice, psychological support, and compliance with medication. • Phone interview 1/week.	LVEF	0.315
	(15.4%)							
(Abtahi et al., 2017) Iran,	25	25	53.6-53.76	8-week	Post-PCI patients	• Three sessions/week, 1hour Each. • A warm-up (10 minutes), and 40 minutes of aerobic exercise training,10 minutes cooling down and finished by relaxation. • 40-60% of HRmax. • 2- Educated about risk factors, stress reduction methods, smoking cessation, body weight control, psychological, and nutritional.	LVESD	0.004
	(42%0						LVEDD	>0.05
							LVEF	<0.001
(Yu et al., 2004) Hong Kong,	181	88	63.7-63.9	8-week	Patients after AMI or elective PCI	Three phases: • Phase 1: inpatient ambulating program lasted for 7 to 14 days. • Phase 2: twice-weekly outpatient exercise and education program lasting for 8 weeks, 1 hour of education class followed by 2 hours of exercise training. • Phase 3: home exercise program lasted 6 months consisted of daily walking exercise for at least 60 minutes after warm-up exercise.	LVESD	˂0.05
	(25%)						LVEDD	ns
							LVEF	ns
(Oliveira et al., 2014) Portugal.	39	44	54.6-58.6	8-week	Patients after AMI	• Three sessions/week 40-minute each (10 minutes of warm-up, 30 minutes of aerobic exercise on a cycle ergometer or treadmill). • 70%-85% of HRmax. • Regular appointments with a cardiologist. • medication optimization.	Vo _2peak_	0.024
	(16.4%)							
(Noites et al., 2017) Portugal	16	16	62.5-59.5	8-week	Patients after AMI	• three sessions/week. 70-85 minutes each include warm-up and cool down. • 60-70 % of HRmax. • moderate-intensity workout.	Vo _2peak_	0.02
	(21.9%)							
(Zhao et al., 2022) China	30	29	60.95	12-week	Patients with CAD	• Six to seven sessions/week, 40–60 minute each. • Included upper and lower extremities, breathing, self-massage, and whole-body exercises. • Moderate-intensity	Vo _2peak_	<0.05
	(71.2%)							
(Kulcu et al., 2007) Turkey	23	21	85.3-60.4	8-week	Patients with CAD	• Three sessions/week, 1 hour each. • Treadmill walking with adjusted speed and inclination. • 60-70 % of HRmax. • Every training session began and ended with 5 min warm-up cool-down periods.	Vo _2peak_	0.001
	(27.2 %)						Anxiety	0.034
							Depression	0.004
(Soleimannejad et al., 2014) Iran.	73	73	58-56.7	8-week	Patients with CAD	• Phase I: (Inpatient): Lasted 7-14 days, initiated after PCI. • Phase II (Outpatient Exercise and Education): Conducted three times weekly, lasting 2-3 months. each session comprised one hour of education followed by two hours of exercise training. • Electrocardiography (ECG) monitoring during exercise sessions. • Phase III (Community-based Home Exercise): Implemented between six and twelve months. • Phase IV (Long-term Follow-up): Extended until the end of the second post-PCI year. emphasized the importance of regular exercise and risk factor modification.	LVESD	0.070
	(31.5%)						LVEDD	0.571
							LVEF	0.006
(Cao et al., 2021) China	24	10	61.7	8-week	patients after PCI	• Three sessions/week, 30 minutes each. • Targeted intensity individually determined based on the HR recorded 1 minute before the threshold of anaerobic ventilation during CPET examination (70–80%) of HRmax. • 5 minutes warm-up, Gradual intensity increase over 20 minutes, 5 minutes cool down.	Vo _2peak_	0.009
	(26.4%)							
(Seki et al., 2003) Japan	20	18	69.3-70.1	6-month	Patients with CAD	• Physical exercise and educational components • Warm-Up Exercises (Approximately 20 Minutes) • Upright Aerobic and Dynamic Exercise (20–30 Minutes) • Cool-Down (Approximately 20 Minutes) • Dietary and Educational Program.	Vo _2peak_	<0.05
	(0%)						Anxiety	<0.01
							Depression	NS
(Ma et al., 2020) China	150	150	63.1-62.8	12-month	Patients post- CABG	• CAD-related health education (Once a week (2 months), each lasting about 60 minutes, covering basic CAD knowledge and post-discharge rehabilitation management. • Exercise guidance and surveillance (once a month for 10 months), 60 to 90 min each time; (3) frequency: 3 to 5 times per week; (4) intensity: fairly light to somewhat hard (11–13 on Borg scale). • Risk factor control (once a month for 10 months). • Psychological nursing (once a month for 10 months).	Anxiety	<0.001
	(21.3%)						Depression	<0.001
(Basati et al., 2012) Iran	15	14	51.7-59	8-week	Patients Post-PCI	• Three session/week, 60-90 minutes each and included 10-20 minutes of warm-up followed by 20-40 minutes of aerobic exercise and a final 10-minute cool-down. In addition, there was a 20-minute relaxation at the end of each session. • 60-85% of HRmax. • (HR) achieved on the exercise test. • Psychological, nutritional, and smoking cessation consultations.	LVEDD	0.001
	(0%)						LVESD	NS
							LVEF	0.001
(Peixoto et al., 2015) Brazil	45	43	56.8-56	30-day	Patients Post-PCI	*Stage I: Early Mobilization Post-AMI* • Begins 12 hours after AMI, conducted twice daily during the inpatient phase. • Starts at 2 METs and progressively increases to 4 METs. • Educational Program covers information about cardiac diagnosis, sexual activity, exercise continuation, nutritional advice, and pharmacological regimen adherence. *Stage II: Unsupervised Progressive Exercise* • 4 times a week for 4 weeks following hospital discharge. • Endurance Training Period: • First week: Starts with a walking time of 20 minutes. • Second week: Increases to 25 minutes. • Third week: increases to 30-35 minutes. • Fourth week: Reaches 35-40 minutes. • Cool-Down Period (5 minutes).	6MWD	<0.001
	(29.5%)							
(Ma et al., 2021) China	70	70	65.2/65.1	12-month	Patients post-CABG	• Health Education: once weekly focusing on topics such as post-CABG medicine management, risk factor management, and secondary prevention. • Rehabilitation Guidance: Includes aerobic, resistance, flexibility, and balance training guidance delivered weekly through video courses. • Exercise Supervision: Individualized exercise prescriptions based on pre-discharge evaluations • Psychological Care: twice weekly personal nurse-led counseling sessions via video call to address mental health and emotional support.	Anxiety	0.034
	(21%)						Depression	0.048
(Højskov et al., 2019) Denmark	152	158	65-65.1	4-week	Patients post-CABG	*Physical Exercise Component* • At admission: Included respiratory physiotherapy and aerobic training. • Post-hospital discharge (up to 4 weeks post-CABG): Involved continuous daily walking, muscle strengthening, and • endurance exercises. *Psycho-educational Component* • Mindfulness practices were a key element, offered as a set of recorded meditation instructions.	Anxiety	0.2
	(14%)						Depression	0.04
(Dwiputra et al., 2023) Indonesia,	44	43	56.3-57.2	6-week	Patients post- CABG	Resistance Training: 2-3 sessions/week Aerobic Training: Participants walked as quickly as possible for 6 minutes on a 45-meter walkway, with allowances to slow down, stop, and rest as needed.	6mwt	0.033
	11%							
(Nishitani-Yokoyama et al., 2018) Japan	18	14	58-59	8- month	Patients with CAD	• Warm-Up: Begins with stretching exercises to prepare muscles and joints. • Aerobic Exercise: Includes cycling, treadmill walking, and in-room track walking for about 20 minutes. • Intensity tailored to individual anaerobic threshold or Borg’s scale rating. • Cool Down: Follows aerobic exercise to gradually lower heart rate. • Schedule: Conducted once daily in the hospital. • Additional Components: • Dietary Recommendations: Follow American Heart Association's phase II diet. • Educational Program: Provided by physicians, nurses, and dietitians, covering CAD and risk factors.	Vo _2peak_	NS
	(6%)							
(Ghashghaei et al., 2012) Iran.	17	15	62-58.5	2-month	Patients post- CABG	• a combination of aerobic and resistance exercise training. • 24 supervised sessions, 90 minutes each, • intensity was set between 60-85% of maximum heart rate. • The program also offered nutritional and psychological consultations, along with risk factor management. • Center vs usual	6-MWD	<0.01
	(19%)						LVESD	0.02
(Oerkild et al., 2012) Denmark	19	21	76.5-77.3	12- month	Patients with CAD	• two visits from a physiotherapist over 6 weeks with a follow-up call for any questions. • 6 sessions/week.30-minute each, with warm-up and cool-down, done Exercises matched personal abilities. • moderate effort (Borg scale 11–13). • Shorter exercises for very disabled patients, done more times a day. • physician visits at the start, 3, 6, and 12 months, plus follow-up calls at 4 and 5 months for monitoring and motivation. • dietary advice and help to quit smoking if needed.	6-MWD	NS
	(43%)							
(Golbus et al., 2023) USA	111/	109	59.5-59.7	6-month	Patients with CAD	• Two types: activity messages and exercise planning messages. • Activity messages encouraged low-level physical activity • Exercise planning messages encouraged participants to exercise the next day • Mobile Application Features: Allowed participants to engage in activity self-monitoring and goal-setting. • Smartwatch data shared with exercise physiologists via email and a web-based dashboard.	6-MWD	0.28
	(30%)							

CAD: coronary artery disease; CAPG: coronary artery bypass graft; PCI: percutaneous intervention; LVEF: left ventricular ejection fraction,

LVEDD: left ventricular end-diastolic dimension, LVESD: left ventricular end-systolic dimension; NP: not provided; NS; not significant;

Vo _2peak_: peak oxygen consumption; HRmax: maximum heart rate; PCI: percutaneous intervention; 6-MWD: six-minute walk distance.

### Risk of bias in included studies

**Figure 2.**
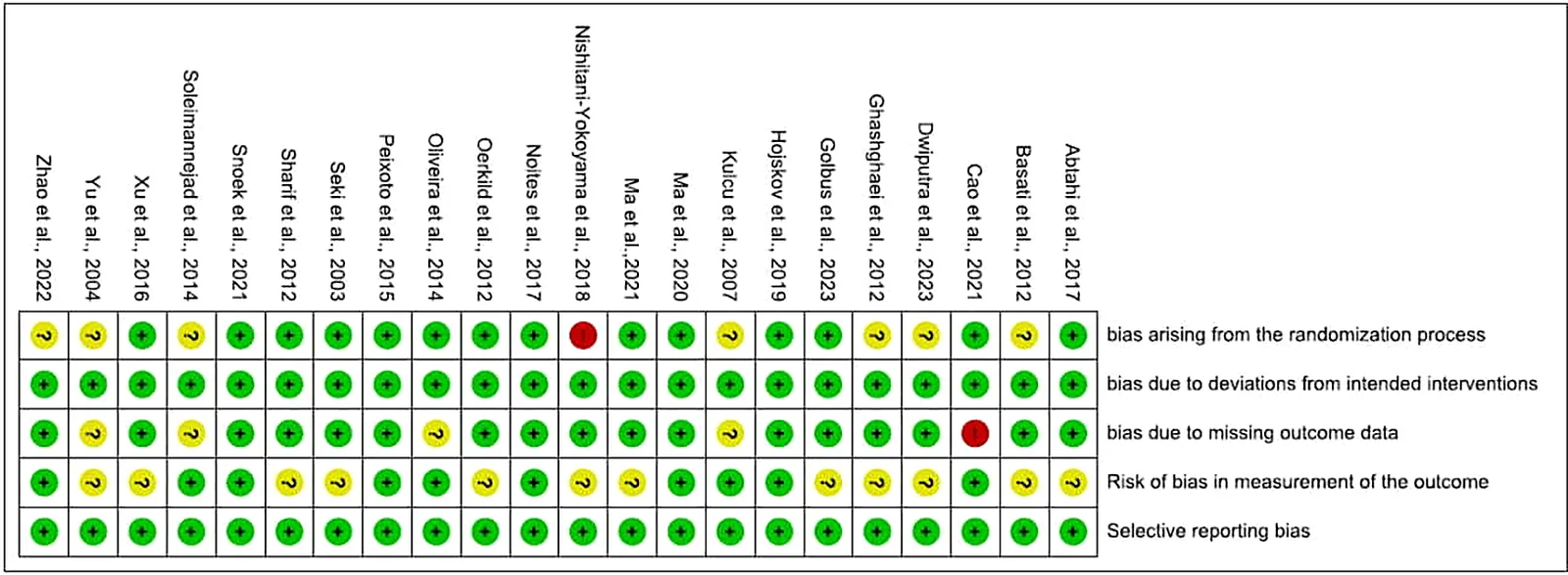
Risk of bias summary of included studies.

**Figure 3.**
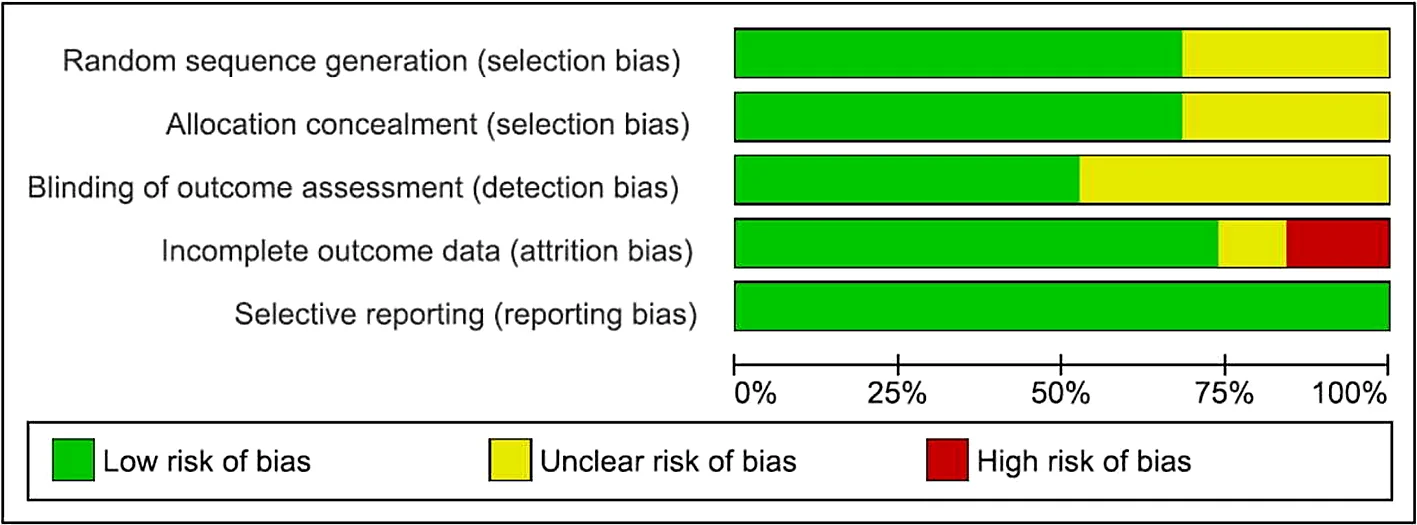
Risk of bias graph.

Of the 22 studies included in this review, 1 study (4.5%) was assessed as having a high risk of bias related to the randomization process. Because the participants were allocated based on the initial assessment. 32% (7 studies) were assessed as having unclear risk of bias since they did not provide information about allocation concealment, the remaining 63% (14 studies) detailed explicitly the allocation methods such as using an opaque envelope, coin flipping, computer-generated numbers and block size. There was no evidence indicating deviation from the intended intervention, therefore, all studies were assessed as having a low risk of bias regarding this domain. regarding missing outcome data, 1 study (4.5%) showed high risk of bias due to a substantial amount of missing data, 18% (4 studies) had an unclear risk of bias, and the remaining 17 studies (77%) reported handling missing data based on intention-to-treat (ITT) analysis. For measurement of the outcome domain, 55% (12 studies) did not provide sufficient information whether the data was analysed blindly or not, resulting in unclear risk of bias. On the other hand, a low risk of bias was observed in the remaining 45% (10 studies), either by reporting blind assessors or by showing a robust methodology. All studies showed low risk regarding reporting bias. Figures 2 and 3 summarize the outcomes of risk bias assessment.

### Certainty of evidence

The GRADE framework was used to evaluate the quality of the evidence in the included studies. Of the seven outcomes investigated in this review, five outcomes had moderate quality evidence (anxiety, depression, VO _2peak_, 6-MWT, and LVEF), and two had low-quality of evidence LVEDD and LVESD. Table 2 details the assessment of certainty of evidence.

**Table 2.** Certainty of evidence.

Outcome	No. studies	No. participants		Risk of bias	Inconsistency	Indirectness	Imprecision	Publication bias	Certainty
		CR	UC						
Anxiety	8	1068		Not serious	Not serious	Not serious	Not serious	undetected	⨁⨁⨁◯ Moderate
		532	532						
Depression	8	1068		serious	Not serious	Not serious	Not serious	undetected	⨁⨁⨁◯ Moderate
		532	534						
VO _2 peak_	8	440		Not serious	Not serious	Not serious	Not serious	undetected	⨁⨁◯◯ Low
		228	212						
6-MWD	5	404		Not serious	Not serious	Not serious	serious	undetected	⨁⨁⨁◯ Moderate
		202	202						
LVEF	6	578		serious	Not serious	Not serious	Not serious	undetected	⨁⨁⨁◯ Moderate
		337	2241						
LVEDD	4	494		Not serious	Not serious	Not serious	Not serious	undetected	⨁⨁⨁◯ Moderate
		294	294						
LVESD	4	494		serious	Not serious	Not serious	serious	undetected	⨁⨁◯◯ Low
		294	200						

CR: cardiac rehabilitation, LVEF: left ventricular ejection fraction, LVEDD: left ventricular end-diastolic dimension, LVESD: left ventricular end-systolic dimension, peak oxygen consumption, 6-MWD: six-minute walk distance, UC: usual care.

## Meta-analysis of included studies

### Psychosocial risk factors

**Figure 4.**
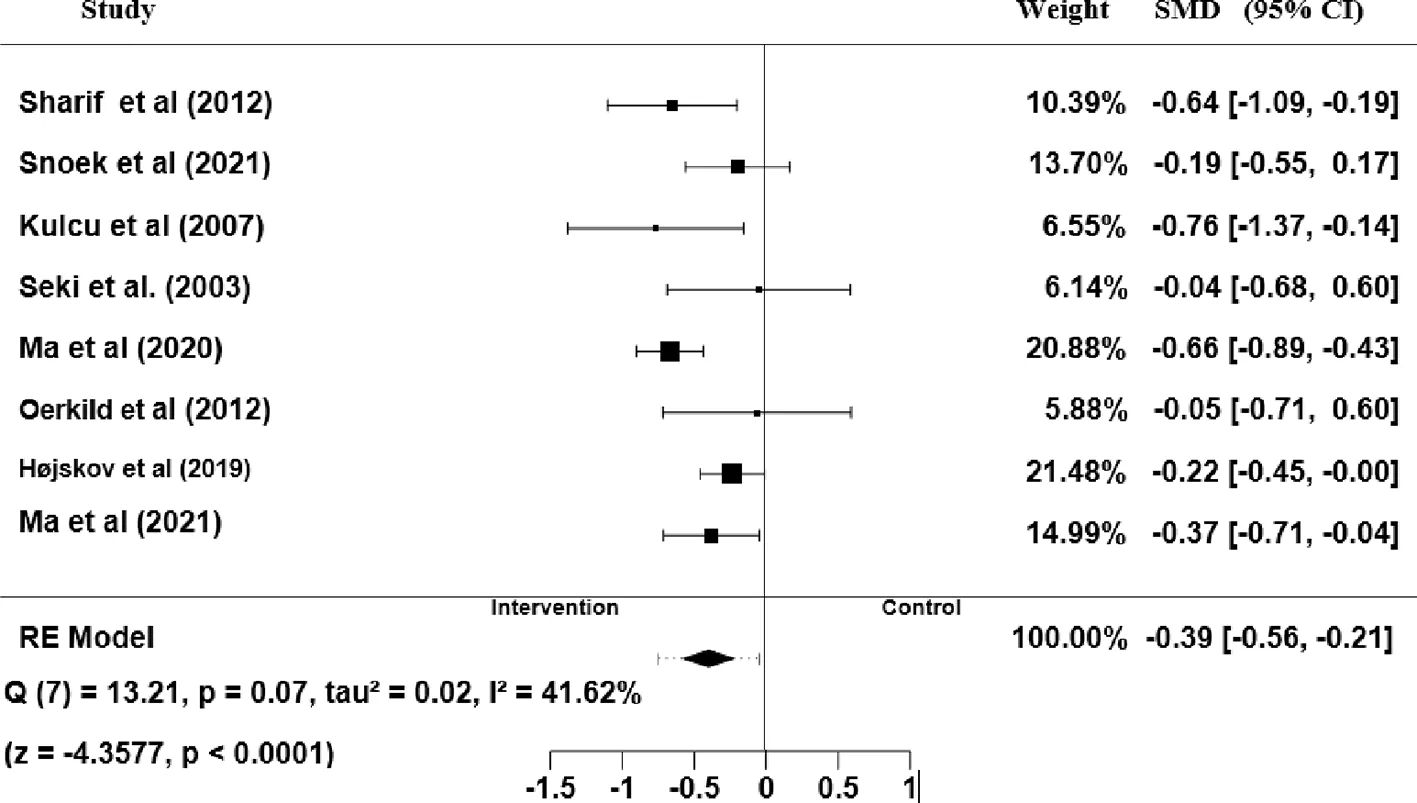
The forest plot of the effect of CR vs usual care on Anxiety.

**Anxiety**: Eight studies evaluated the effect of CR on anxiety levels. Meta-analysis showed a statistically significant reduction in anxiety levels in intervention groups compared with control SMD = -0.3890 (95% CI: -0.5640 to -0.2140). (z = -4.3577, p < 0.0001). There was a moderate amount of heterogeneity in the true outcomes, I ^2^ = 41.62%, p = 0.0672. No indication of publication bias was observed (see Figure 4).

**Figure 5.**
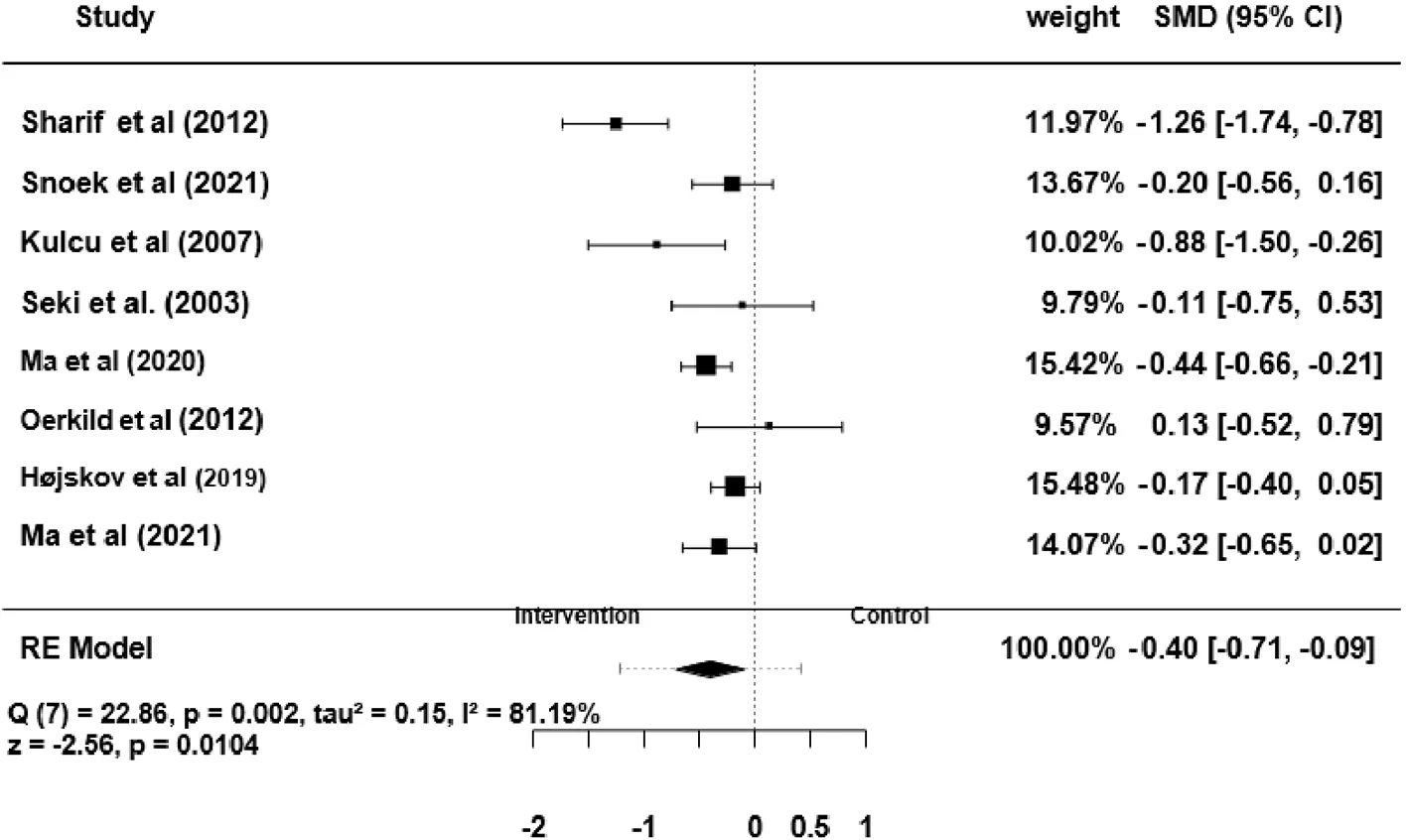
The forest plot of the effect of CR vs usual care on Depression.

**Depression**: Eight studies examined the effect of CR on depression. Meta-analysis revealed a statistically significant reduction in depression levels among intervention groups compared to control. SMD = -0.4032 (95% CI: -0.7114 to -0.0950). (z = -2.5638, p = 0.0018). However, a high amount of heterogeneity was observed I ^2^ = 81.1861%, p = 0.0034. employing the sensitivity test by removing the study by Sharif et al. (2012) ^33^ and the study by Kulcu et al. (2007) ^32^ revealed statistically significant result without a significant amount of heterogeneity. SMD = -0.2663 (95% CI: -0.3948 to -0.1378), (z = -4.0626, p < 0.0001). I ^2^ = 0.0000%, p = 0.4598. No indication of publication bias was observed (see Figure 5).

### Functional capacity

**Figure 6.**
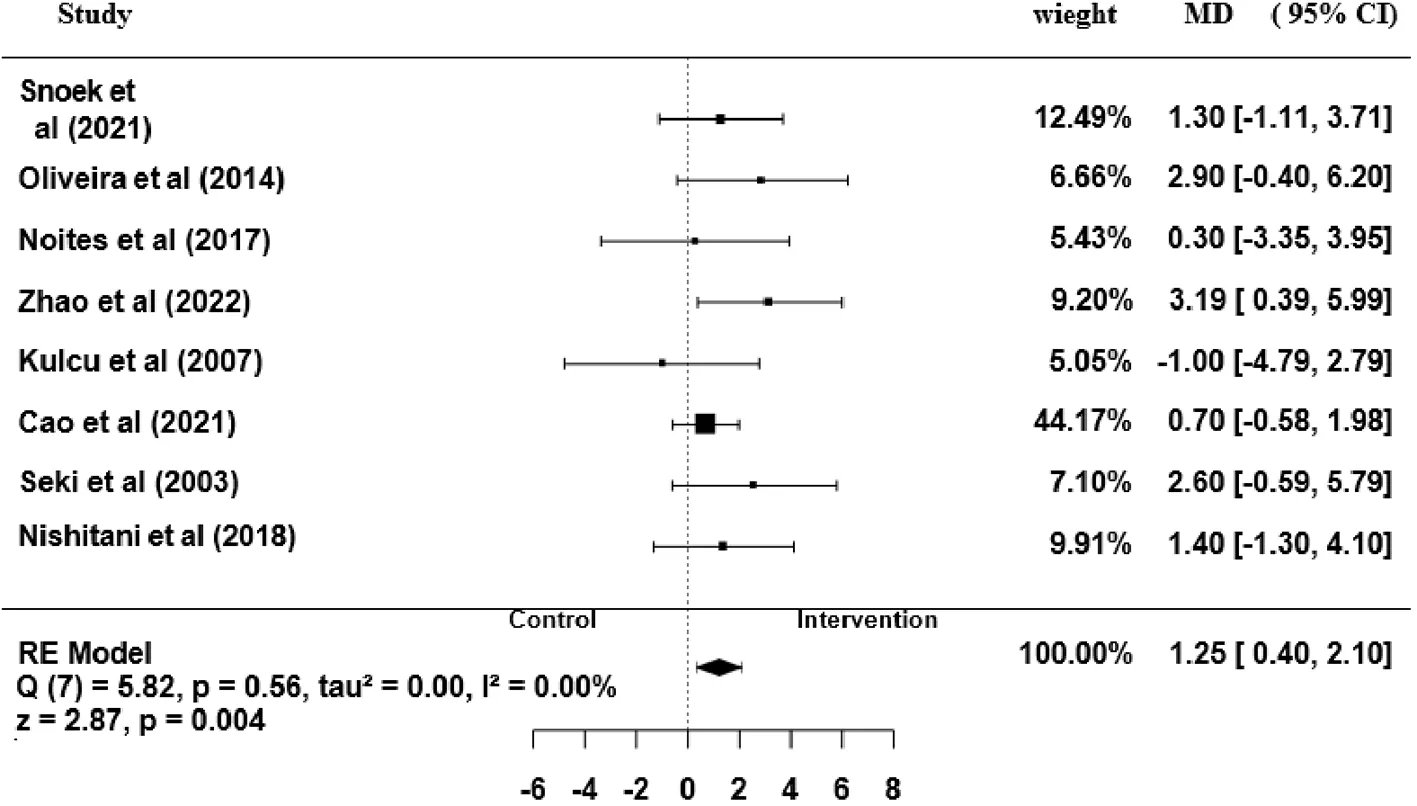
The forest plot of the effect of CR vs usual care on VO _2_ peak.

**Peak VO**_**2peak**_ **:** Eight studies investigated the impact of CR on VO _2_ peak. Meta-analysis showed a statistically significant improvement in Peak VO _2_ among participants in intervention groups compared to those in control groups. MD = 1.2471 (95% CI: 0.3963 to 2.0979). (z = 2.8728, p = 0.0041). There was no significant amount of heterogeneity in the true outcomes I ^2^ = 0.0000%. p = 0.5604. No indication of publication bias was observed (see Figure 6).

**Figure 7.**
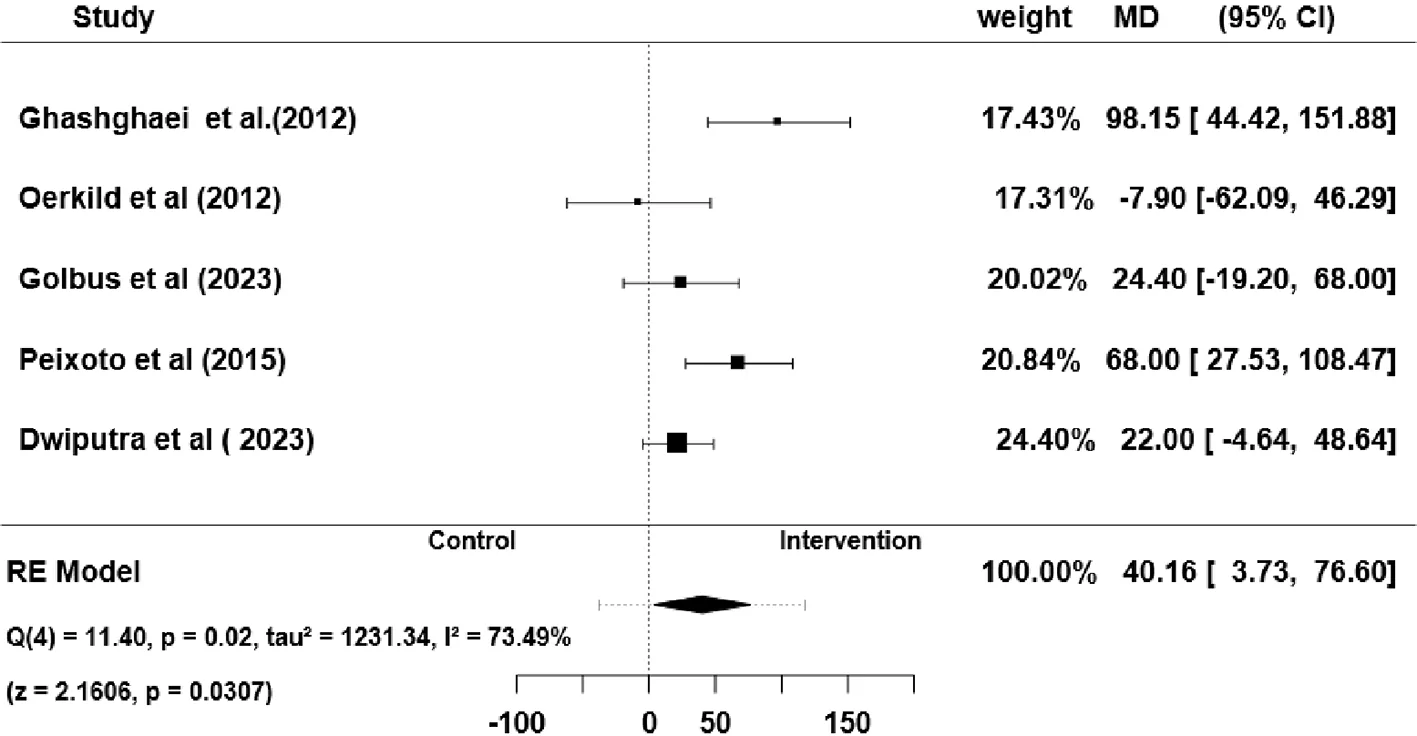
The forest plot of the effect of CR vs usual care on 6-MWT.

**6-MWT:** Five studies investigated the effect of CR vs usual care on 6-MWT. The pooled effect showed a statistically significant increase in 6-MWT among participants in intervention groups compared to those in control groups. MD = 54.7948 (95% CI: 15.6115 to 93.9782). (z = 2.1630, p = 0.0305). However, the true outcomes appear to be heterogeneous, I ^2^ = 73.3622%, p = 0.0234. employing the sensitivity test by removing the study by Oerkild et al. (2012) ^36^ and the study by Ghashghaei et al. (2012) ^35^ revealed a statistically significant result MD = 36.0290 (95% CI: 7.7497 to 64.3082). (z = 2.4971, p = 0.0125) with moderate amount of heterogeneity, I ^2^ = 45.3613%, p = 0.1670. No indication of publication bias was observed (see Figure 7).

### Left ventricular function

**Figure 8.**
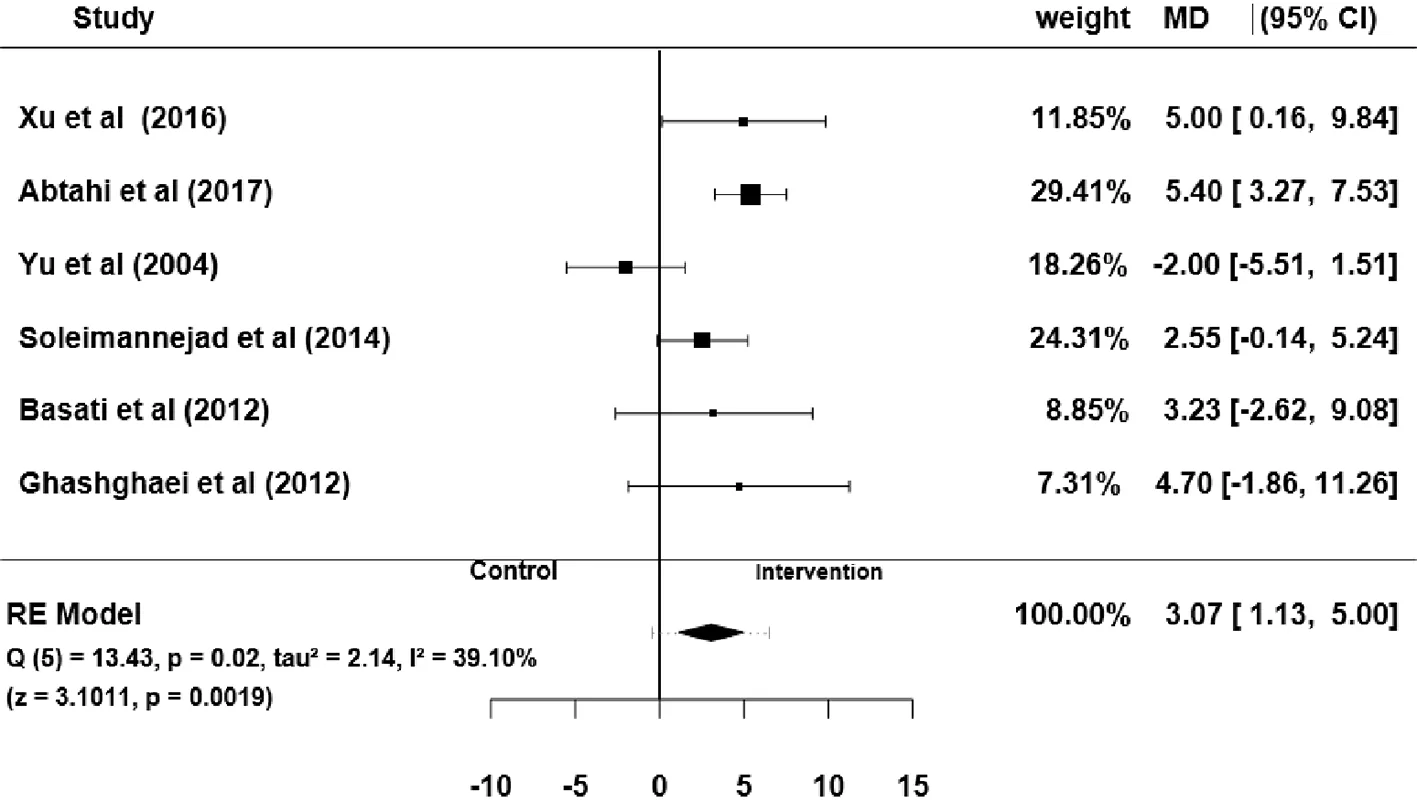
The forest plot of the effect of CR vs usual care on LVEF.

**LVEF**: Six studies examined the effect of CR vs usual care on LVEF. When compared to the control, participants in intervention groups demonstrated a statistically significant increase in LVEF. MD = 3.0650 (95% CI: 1.1279 to 5.0022). (z = 3.1011, p = 0.0019) with a moderate amount of heterogeneity, I ^2^ = 39.10%, p = 0.0196. No indication of publication bias was observed (see Figure 8).

**Figure 9.**
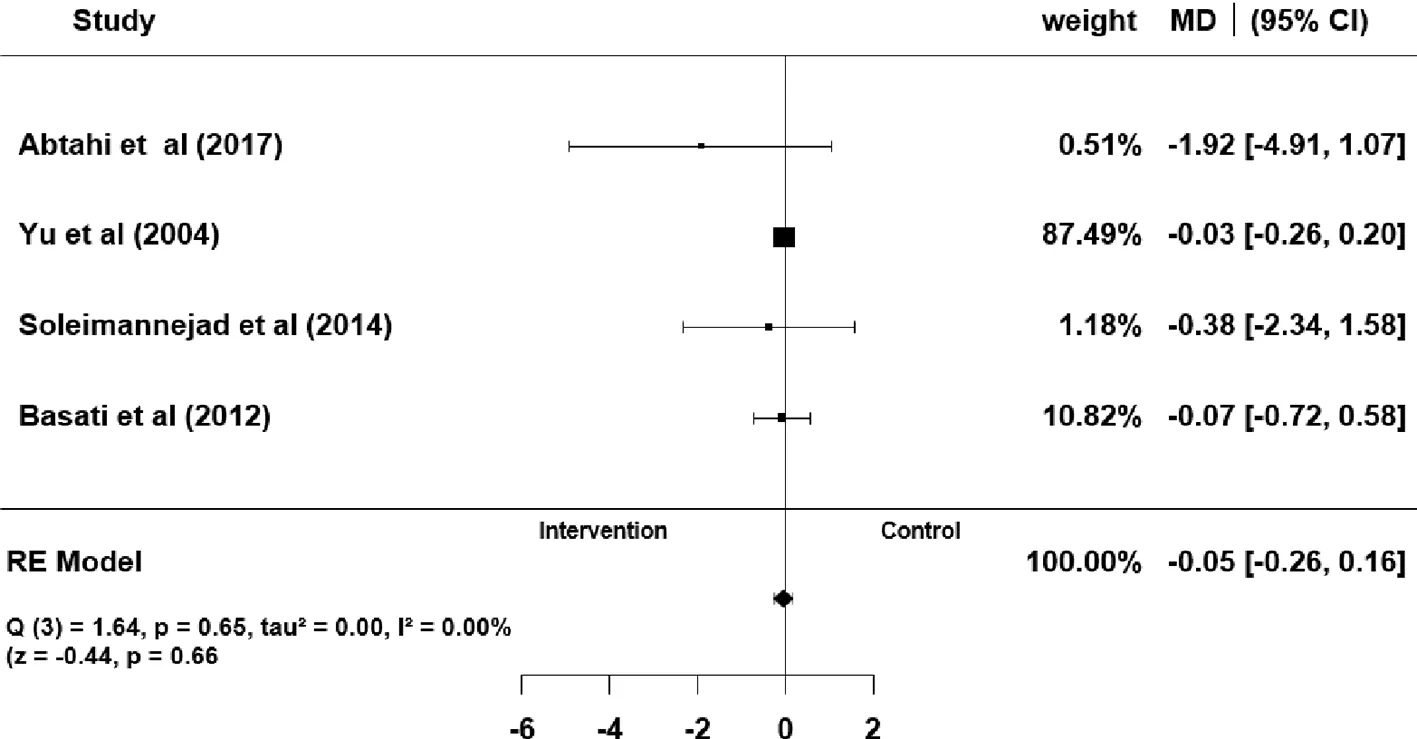
The forest plot of the effect of CR vs usual care on LVEDD.

**LVEDD:** Four studies evaluated the impact of CRP vs usual care on LVEDD. Meta-analysis demonstrated a non-statistically significant reduction in LVEDD in intervention groups compared to control, = -0.0480 (95% CI: -0.2609 to 0.1648). (z = -0.4422, p = 0.6583). and the heterogeneity was null I ^2^ = 0.0000%, p = 0.6501. No indication of publication bias was observed (see Figure 9).

**Figure 10.**
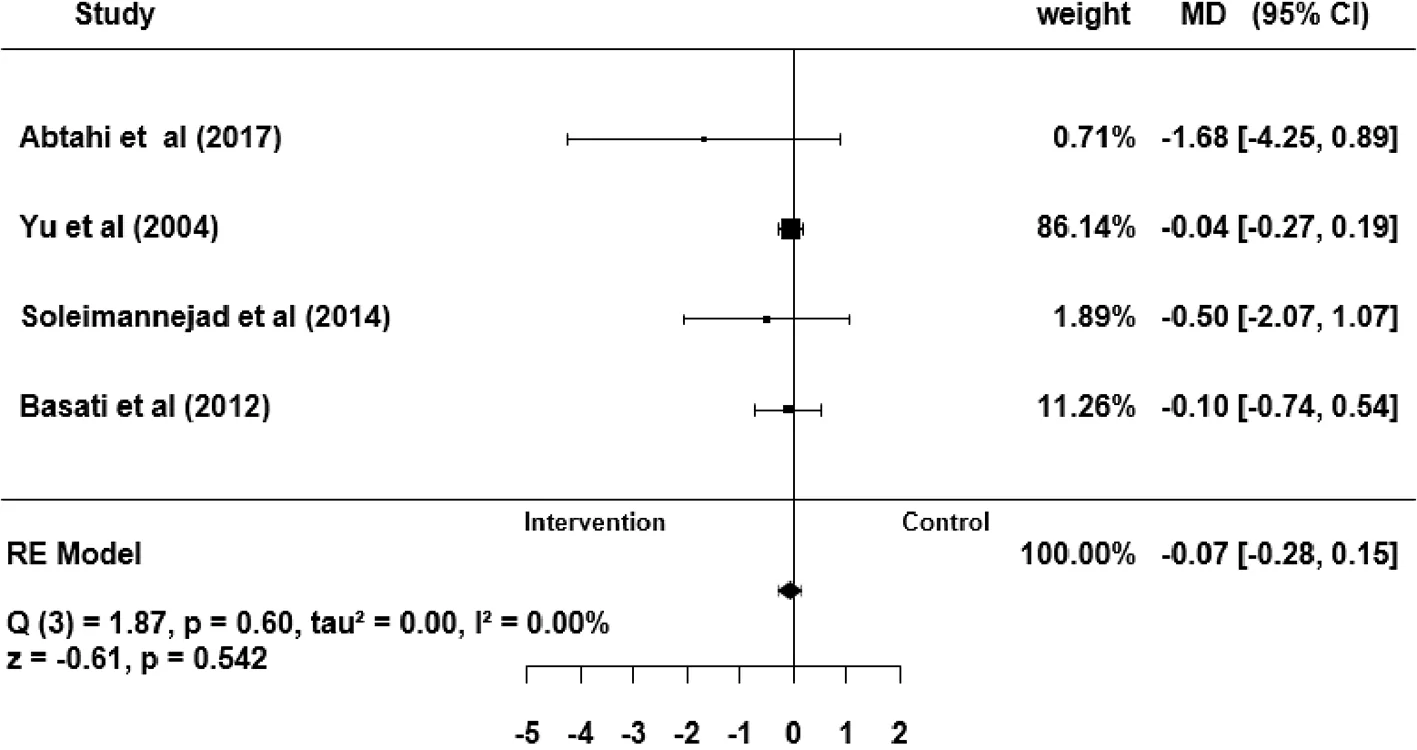
The forest plot of the effect of CR vs usual care on LVESD.

**LVESD:** Five studies assessed the impact of CRP vs usual care on LVESD. Meta-analysis revealed that the intervention groups exhibited a non-statistically significant decrease in LVESD compared to control groups. MD = -0.0670 (95% CI: -0.2829 to 0.1489). (z = -0.6086, p = 0.5428). All studies appeared homogeneous I ^2^ = 0.0000%, p = 0.6005. No indication of publication bias was observed (see Figure 10).

## Discussion

The main findings for this systematic review and meta-analysis were that CR improved psychosocial risk factors, functional capacity, and left ventricular function, as indicated by the statistically significant improvement in (anxiety, depression, VO _2peak_, 6-MWT, and LVEF) in intervention groups as compared to control, and the favourable trend toward intervention groups in LVEDD and LVESD. There was no significant amount of heterogeneity among all outcomes, except for depression, 6-MWT, and LVEF. However, the heterogeneity could be handled by sensitivity analysis.

Constant feelings of fear and worry about social and physical aspects of life influence the patient’s mental health. ^51^ Anxiety and depression are common psychological disorders after cardiovascular events such as acute myocardial infarction and revascularization. ^51^^,^^52^ Studies documented that the prevalence of anxiety and depression in patients diagnosed with CAD is very high compared to the general population. ^53^^,^^54^ A recent meta-analysis aimed to estimate the prevalence of anxiety and depression in cardiac patients reported that the prevalence of anxiety was 32.9% (95% CI: 21.9-46.6%), while depression was noted at 31.3% (95% CI: 21.9-46.6%). ^55^

Those cardiac patients who are anxious and depressed are more susceptible to major adverse cardiac events and death. ^56^^–^^58^ This creates the need for early assessment and treatment of anxiety and depressive symptoms. The findings of this review emphasize the ability of CR to alleviate anxiety and depression. By training the patients to adhere to a healthy lifestyle, CR aims to enhance patients’ satisfaction and return to normal life. Multidisciplinary CR that include physical training, psychological support, and health education appeared to be highly effective and have shown significant benefits in reducing anxiety and depression levels. The result of this review was in line with previous works. For instance, Yohannes and colleagues concluded that CR had a positive impact on particular psychological disorders, such as depression and anxiety and this impact was maintained at short and long follow-up. ^59^ Escobar and colleagues also observed improvement in anxiety and depressive symptoms in patients experiencing CR. ^60^

Functional capacity is a major outcome of CR, considered a key element in CAD secondary prevention. ^61^ Functional capacity adversely affected by CAD results in a sedentary lifestyle. ^62^^,^^63^ The inverse correlation between functional capacity and mortality rate has been reported in many studies. In a study conducted by Laukkanen and colleagues aimed to evaluate the relationship between functional capacity and cardiac and all-cause mortality rate, functional capacity appeared as a strong predictor of mortality beyond traditional risk factors. ^64^

Functional capacity is frequently evaluated by VO _2peak_ and 6-MWT. ^65^ VO _2peak_ is a vital physiological indicator determining an individual’s upper capability for oxygen utilization during strenuous physical exertion, ^66^ as this metric reflects cardiovascular fitness and aerobic endurance it plays a crucial role in shaping exercise prescription and assessing the efficiency of therapeutic interventions such as CR. ^67^ Additionally, the 6-MWT provides a practical and valuable evaluation of functional capacity. serves as a fundamental marker of cardiovascular fitness and aerobic endurance. ^65^^,^^68^

The present review demonstrated significant improvement in functional capacity in CR groups compared to usual care. While the heterogeneity in the VO _2peak_ outcome was low, it was high in the 6-MWT outcome, after employing the sensitivity test by removing the studies by Oerkild et al. (2012) ^36^ and Ghashghaei et al. (2012) ^35^ the result remained significant, with moderate heterogeneity. The relatively smaller sample sizes of these two studies compared to other studies in this outcome may contribute to the heterogeneous result observed. These significant results highlight the effectiveness of CR in enhancing patients’ functional capacity and overall health. These findings are supported by the findings of a recent scientific review, where the authors observed improvement in functional capacity after CR. ^69^^,^^70^

Despite the advancement in the contemporary treatment aimed at restoring coronary blood flow, the injury caused by myocardial ischemia leads to left ventricular remodelling; a transformative process that involves changes in the left ventricle’s dimensions, shape, and function. ^25^ Therefore, the need for a treatment that enhances the left ventricular function without adversely affecting the ventricular remodelling is essential.

The findings of the present review demonstrated that CR significantly improved the left ventricular function. as evidenced by the significant increase in LVEF and the reduction in LVEDD and LVESV in intervention groups as compared with the control. Although the reduction in LVEDD and LVESD between the intervention and the control groups was not statistically significant, a favourable trend was observed in the participants in intervention groups without heterogeneity. These results emphasize that CR did not adversely affect the structure, shape, and function of the heart. These findings are consistence with the findings of previous studies which conclude that CR has favourable effects on LV function and remodelling in patients with CAD. ^71^^–^^73^

Some limitations in this review should be mentioned. Firstly, the variation of CR in terms of onset, duration, intensity and frequency among the included studies may influenced the outcomes differently, CR with higher does may produce better improvement. Secondly, the small sample size in some included studies diminished the statistical power and increased the heterogeneity in particular investigated outcomes. Thirdly, some studies lacked sufficient information to allow for judgment on the risk of bias, especially information about blinding of the data assessors. Finally, the limited number of available trials that met the inclusion criteria especially those that investigated the impact of CR on 6-MWT, LVEDD and LVESD. Future studies are recommended to explore the impact of CR on other physical, psychological, and cardiac outcomes, such studies may comprehensively clarify the beneficiary effect of CR and underscore the areas of improvement.

## Conclusion

For patients with Coronary Artery Disease, including patients after PCI or CABG, cardiac rehabilitation effectively improved psychosocial risk factors such as anxiety and depression, functional capacity as measured by VO _2peak_ and 6-MWT, and left ventricular ejection fraction. The findings underscore CR as a method that enhances patients’ overall health.

## Data availability

### Underlying data

“All data underlying the results are available as part of the article and no additional source data are required.”

## Software and code

The Endnote reference manager available from: https://endnote.com/downloads/ alternative free access reference manager available from: https://www.mendeley.com/download-reference-manager/windows

Meta-analysis statistical spreadsheet available from: https://www.jamovi.org/download.html

Risk of bias assessment available from: https://sites.google.com/site/riskofbiastool/welcome/rob-2-0-tool/current-version-of-rob-2?authuser=0

## Reporting guidelines

Zenodo: PRISMA Checklist for Article “The Impact of Cardiac Rehabilitation on Psychosocial Factors, Functional Capacity, and Left Ventricular Function in Patients with Coronary Artery Disease: Systematic Review and Meta-Analysis”. https://doi.org/10.5281/zenodo.11186070

Data are available under the terms of the Creative Commons Zero “No rights reserved” data waiver (CC0 1.0 Public domain dedication).

Zenodo: PRISMA flow chart for Article “The Impact of Cardiac Rehabilitation on Psychosocial Factors, Functional Capacity, and Left Ventricular Function in Patients with Coronary Artery Disease: Systematic Review and Meta-Analysis”. https://doi.org/10.5281/zenodo.11186649

Data are available under the terms of the Creative Commons Zero “No rights reserved” data waiver (CC0 1.0 Public domain dedication).
