# Supplementary material for: The Impact of Cardiac Rehabilitation on Psychosocial Factors, Functional Capacity, and Left Ventricular Function in Patients with Coronary Artery Disease: Systematic Review and Meta-Analysis
Source: F1000Res. 2026 Jun 25;13:575. Originally published 2024 Jun 5. [Version 2] doi: 10.12688/f1000research.151834.2 (PMC13575403; doi:10.12688/f1000research.151834.2)
Supplement: Peer review history

# Reviewer report: Reviewer response for version 2

**DOI:** 10.5256/f1000research.201135.r515667

**Published:** 2026-09-16

**Authors:** Mehwish Waseem, Azka Batool

**Affiliations:** 1 Riphah International University, Islamabad, Pakistan; 2 Rehabilitation, Riphah International University Islamic International Medical College, Rawalpindi, Punjab, Pakistan

A minor improvement would be to make the evidence gap more explicit. The introduction states that cardiac rehabilitation research is dynamic and new evidence needs to be synthesized but it could more clearly state what limitations of previous systematic reviews/meta-analyses led to the present review and what new contribution this review makes. This would provide additional justification to perform another systematic review.

 Methods are generally appropriate and sufficiently described to understand the overall methodology. Further information is needed to ensure full reproducibility. The authors should report the complete database-specific search strategies and dates, specify the eligible types and characteristics of cardiac rehabilitation, ensure consistency between the abstract and Methods regarding the databases searched, provide more details regarding the meta-analytic procedures, and reconcile the RoB 2 terminology used in the Methods and Results. The rationale for downgrading evidence using GRADE should also be reported explicitly for each outcome.

 The statistical approach is generally appropriate, including the use of a random-effects model, selection of effect sizes, heterogeneity assessment, sensitivity analysis and publication-bias assessment. But the interpretation needs to be revised. In particular, the high heterogeneity found for depression and 6-MWT should be investigated in more depth and the reasons for excluding some studies in sensitivity analyzes should be explicitly mentioned. Authors should differentiate between statistical and clinical significance and not consider non-significant reductions in LVEDD and LVESD as evidence of improvement. More transparent reporting of subgroup analyzes and the impact of risk of bias on pooled estimates are also needed.

 The results of the meta-analysis generally support the conclusions. But the authors should be careful in interpreting the cardiac structural outcomes as LVEDD and LVESD were not statistically different. The conclusions should also take into account the high heterogeneity found for depression and 6-MWT and moderate/low certainty of evidence for some outcomes. The statement that cardiac rehabilitation improves “overall health” is broader than the outcomes directly assessed and should be qualified or limited to the outcomes measured.

Are the rationale for, and objectives of, the Systematic Review clearly stated?

Yes

Is the statistical analysis and its interpretation appropriate?

Partly

If this is a Living Systematic Review, is the ‘living’ method appropriate and is the search schedule clearly defined and justified? (‘Living Systematic Review’ or a variation of this term should be included in the title.)

Not applicable

Are sufficient details of the methods and analysis provided to allow replication by others?

Partly

Are the conclusions drawn adequately supported by the results presented in the review?

Yes

Reviewer Expertise:

Cardiopulmonary Rehabilitation

We confirm that we have read this submission and believe that we have an appropriate level of expertise to confirm that it is of an acceptable scientific standard.

# Reviewer report: Reviewer response for version 2

**DOI:** 10.5256/f1000research.201135.r515668

**Published:** 2026-09-14

**Authors:** Nabeel Al-Yateem

**Affiliations:** 1 University of Sharjah, Sharjah, United Arab Emirates

**The Impact of Cardiac Rehabilitation on Psychosocial Factors, Functional Capacity, and Left Ventricular Function in Patients with Coronary Artery Disease: Systematic Review and Meta-Analysis**

**Reviewer comments**

 This systematic review and meta-analysis address an important clinical topic by examining the multidimensional benefits of cardiac rehabilitation in patients with coronary artery disease. Its systematic search, independent data extraction, and structured assessments of risk of bias and evidence certainty reflect a rigorous approach to evidence synthesis. The conclusions highlight improvements in psychological well-being, functional capacity, and left ventricular ejection fraction, reinforcing the clinical relevance of the findings and the value of cardiac rehabilitation in comprehensive patient care. However, addressing the following minor comments may further improve the manuscript’s clarity, readability, and overall presentation.

- The Introduction could benefit from stronger connections between psychosocial factors, functional capacity, and left ventricular function, clarifying why these outcomes were examined together.
- The intervention descriptions in Table 1 could be slightly shortened to make the table easier to read.
- If possible, it would be beneficial to update the literature search and include any relevant recently published studies.
- The artwork for the risk-of-bias figures and forest plots could be enhanced using modern visualization software.
- It would be more beneficial for the Discussion section to focus further on the clinical implications of the findings.

Are the rationale for, and objectives of, the Systematic Review clearly stated?

Yes

Is the statistical analysis and its interpretation appropriate?

I cannot comment. A qualified statistician is required.

If this is a Living Systematic Review, is the ‘living’ method appropriate and is the search schedule clearly defined and justified? (‘Living Systematic Review’ or a variation of this term should be included in the title.)

Not applicable

Are sufficient details of the methods and analysis provided to allow replication by others?

Yes

Are the conclusions drawn adequately supported by the results presented in the review?

Yes

Reviewer Expertise:

Child and Adolescent Health, Chronic Conditions, Nursig Research.

I confirm that I have read this submission and believe that I have an appropriate level of expertise to confirm that it is of an acceptable scientific standard.

# Reviewer report: Reviewer response for version 2

**DOI:** 10.5256/f1000research.201135.r504578

**Published:** 2026-08-12

**Authors:** Sujeewa Dilhani Maithreepala

**Affiliations:** 1 University of Peradeniya, Kandy, Sri Lanka

**Suggested Reviewer Comments**

1. **Search strategy:** In main body (Please provide keywords used);;;;

as supplementary material - Please provide the complete search strategy for each database, including keywords/MeSH terms, Boolean operators, database-specific syntax, and the date of the last search,

1. **Update the search:** The search covers studies only up to 2024; if possible, please update the literature search to include relevant studies published from **2024 to 2026**.
2. Need to correct the inconsistencies between the text and Table 2. VO₂peak and LVEDD have different certainty ratings in the text and table. Also, please recheck the LVEF participant numbers in Table 2, as they appear inconsistent with the reported total.
3. **Risk of bias:** Please ensure that the terminology and judgments are consistent with **RoB 2** throughout the text, tables, and figures.
4. **Publication bias:** Most analyses include fewer than 10 studies; therefore, publication bias cannot be reliably assessed. Please acknowledge this limitation and use more cautious wording.
5. **Heterogeneity:** Please recheck the heterogeneity statistics, particularly for LVEF, and ensure that **Q, I², and p-values** are reported consistently across the text and figures.
6. **Left ventricular outcomes:** Please revise the conclusion regarding improved left ventricular function. LVEF improved significantly, whereas LVEDD and LVESD did not. The conclusions should clearly reflect these findings.
7. **Language:** The manuscript still contains several grammatical and typographical errors (e.g., “a key an indicator,” “suplly,” “left ventriculare,” “statistical significant,” and “are consistence”). Further language editing is recommended.
8. **Protocol registration:** Please clarify whether the systematic review protocol was prospectively registered (e.g., **PROSPERO**) and provide the registration number, if available.
9. **Table 1:** Table 1 is very detailed and difficult to read. Please consider shortening the intervention descriptions and moving additional details to supplementary material.
10. **Subgroup analyses:** The Methods state that subgroup analyses were conducted by follow-up duration and mean age, but these results are not clearly reported. Please report the subgroup analyses or revise the Methods accordingly.
11. Needs to clearly state the current evidence gap, why this systematic review and meta-analysis is needed, and its novelty compared with previous reviews, supported by relevant references.
12. Definition of cardiac rehabilitation: Please clearly define the cardiac rehabilitation intervention included in this review (e.g., conventional/centre-based, home-based, or internet/digital-based CR) and clearly specify the comparator (usual care/no CR). Ensure these definitions and eligibility criteria are applied consistently throughout the manuscript

Are the rationale for, and objectives of, the Systematic Review clearly stated?

Partly

Is the statistical analysis and its interpretation appropriate?

Yes

If this is a Living Systematic Review, is the ‘living’ method appropriate and is the search schedule clearly defined and justified? (‘Living Systematic Review’ or a variation of this term should be included in the title.)

No

Are sufficient details of the methods and analysis provided to allow replication by others?

No

Are the conclusions drawn adequately supported by the results presented in the review?

Yes

Reviewer Expertise:

Heart Failure, Cardiovascular Health, Cardiac Rehabilitation, and Digital Health.

I confirm that I have read this submission and believe that I have an appropriate level of expertise to confirm that it is of an acceptable scientific standard, however I have significant reservations, as outlined above.

# Reviewer report: Reviewer response for version 2

**DOI:** 10.5256/f1000research.201135.r496560

**Published:** 2026-07-11

**Authors:** Thomas Maribo

**Affiliations:** 1 Department of Public Health, Aarhus University, Aarhus, Denmark

The authors have addressed my previous comments satisfactorily. The manuscript has improved substantially and is scientifically suitable for indexing. I recommend acceptance after minor editorial language editing, as a number of grammatical and stylistic issues remain throughout the manuscript.

Are the rationale for, and objectives of, the Systematic Review clearly stated?

Yes

Is the statistical analysis and its interpretation appropriate?

Yes

If this is a Living Systematic Review, is the ‘living’ method appropriate and is the search schedule clearly defined and justified? (‘Living Systematic Review’ or a variation of this term should be included in the title.)

No

Are sufficient details of the methods and analysis provided to allow replication by others?

Yes

Are the conclusions drawn adequately supported by the results presented in the review?

Yes

Reviewer Expertise:

Please make minor adjustments as suggested

I confirm that I have read this submission and believe that I have an appropriate level of expertise to confirm that it is of an acceptable scientific standard, however I have significant reservations, as outlined above.

# Reviewer report: Reviewer response for version 1

**DOI:** 10.5256/f1000research.166520.r288105

**Published:** 2024-06-29

**Authors:** Thomas Maribo

**Affiliations:** 1 Department of Public Health, Aarhus University, Aarhus, Denmark

The systematic review and meta-analysis explores the impact of cardiac rehabilitation on psychosocial factors, functional capacity, and left ventricular function in patients with coronary artery disease. The study includes data from 22 randomized controlled trials involving 2283 participants, comparing cardiac rehabilitation to usual care. The findings indicate significant improvements in anxiety, depression, peak oxygen consumption, six-minute walk distance, and left ventricular ejection fraction among patients undergoing cardiac rehabilitation. The study underscores the importance of cardiac rehabilitation in enhancing psychological well-being and physical health in patients with coronary artery disease.

 The results are highly interesting in relation to cardiac rehabilitation and care. So far, there has been a strong focus on the physical part of cardiac rehabilitation with an increasing interest in psychosocial factors. This article provides good evidence that there is a need to focus more on the psychosocial factors - without losing focus on the physical factors.

 The article is a good and solid contribution to this transformation

Are the rationale for, and objectives of, the Systematic Review clearly stated?

Yes

Is the statistical analysis and its interpretation appropriate?

Yes

If this is a Living Systematic Review, is the ‘living’ method appropriate and is the search schedule clearly defined and justified? (‘Living Systematic Review’ or a variation of this term should be included in the title.)

No

Are sufficient details of the methods and analysis provided to allow replication by others?

Yes

Are the conclusions drawn adequately supported by the results presented in the review?

Yes

Reviewer Expertise:

Please make minor adjustments as suggested

I confirm that I have read this submission and believe that I have an appropriate level of expertise to confirm that it is of an acceptable scientific standard.

## References

[1] : Cardiac Rehabilitation - Challenges, Advances, and the Road Ahead. *N Engl J Med* .2024;390(9) : 10.1056/NEJMra2302291830-841 10.1056/NEJMra2302291 38416431
